# Immune regulation of metastasis: mechanistic insights and therapeutic opportunities

**DOI:** 10.1242/dmm.036236

**Published:** 2018-10-24

**Authors:** Olga S. Blomberg, Lorenzo Spagnuolo, Karin E. de Visser

**Affiliations:** Division of Tumor Biology and Immunology, Oncode Institute, The Netherlands Cancer Institute, Plesmanlaan 121, 1066 CX Amsterdam, The Netherlands

**Keywords:** Metastasis, Inflammation, Immunotherapy, (Pre-)metastatic niche

## Abstract

Metastatic disease is the leading cause of death in cancer patients. Metastasis formation involves a cascade of events for which the underlying mechanisms are still poorly understood. During the metastatic cascade, cancer cells tightly interact with the immune system and they influence each other, both in the tumor microenvironment and systemically. The crosstalk between cancer and immune cells adds another layer of complexity to our understanding of metastasis formation, but at the same time opens new therapeutic opportunities for cancer patients. The intensifying development of immunotherapeutic strategies calls for a better understanding of immune regulation of metastasis in order to maximize the therapeutic benefit for patients with metastatic disease. In this Review and accompanying poster, we describe the main mechanisms of immune regulation of metastasis that have been reported to date, and present promising immunotherapeutic options that are currently available, or may become so in the near future, to tackle metastasis.

## Abstract (precis)

**Summary:** In this Review and accompanying poster, we discuss the dual role of the immune system in metastasis and summarize promising therapeutic strategies to target tumor-immune interactions.

## The dual role of the immune system in cancer

Cancer is one of the leading causes of death worldwide, and the vast majority of cancer-related deaths are a consequence of metastasis. Patients with advanced metastatic disease are, with rare exception, incurable by current treatment options. The process that leads to metastasis is referred to as the metastatic cascade (see poster). Briefly, to escape from the primary tumor site, cancer cells have to invade the surrounding tissue and intravasate into blood or lymphatic vessels, from which they can then spread throughout the body. Metastasis results from subsequent cancer cell extravasation at distant sites, followed by successful local outgrowth.

**Figure:**
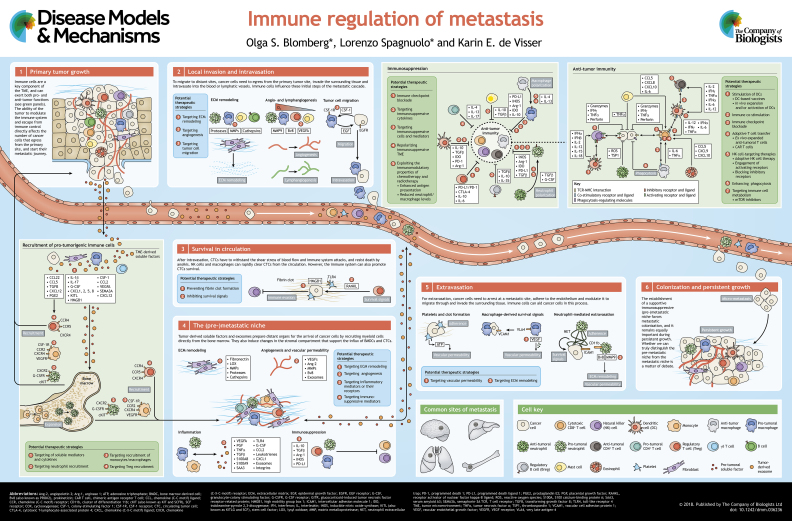

Throughout metastasis formation, cancer cells interact with the immune system, which can modulate each step of the cascade. The notion of a close interplay between tumor and immune cells dates back to the 19th century, when Rudolf Virchow first observed leukocytes infiltrating malignant tissues and hypothesized that cancers originate from chronically inflamed sites (Balkwill and Mantovani, 2001). Conversely, the immune system's anti-tumor potential was first postulated at the end of the same century by William Coley, who noticed that a patient with an inoperable, recurring sarcoma was completely cured after a concurrent infection. Inspired by this case, he treated cancer patients with a mixture of killed bacteria, which later became known as Coley's toxin (Coley, 1893), obtaining impressive clinical results. Coley's toxin was indeed the first cancer immunotherapy approach in history. These early observations suggest how, in the context of cancer, the immune system plays a dual role, and that a tumor's fate depends on the balance between anti-tumor immunity and tumor-promoting inflammation.

Besides shaping the development and outgrowth of primary tumors (see poster, panel 1, ‘Primary tumor growth’), the immune system influences various steps of the metastatic cascade. In renal cell carcinoma patients, for instance, circulating monocytes have pro-metastatic functions. Compared with monocytes from healthy donors, patient-derived monocytes showed increased capability to promote cancer cell invasion and angiogenesis (Chittezhath et al., 2014). In colorectal cancer patients, the infiltration of T cells in the primary tumor was associated with reduced probability of tumor dissemination, suggesting an anti-tumor role of the adaptive immune system in the early stages of the metastatic process (Pagès et al., 2005). Although research revealed some of the metastasis-modulating effects of the immune system, the complexity of metastasis formation and the scarcity of accurate tumor models that properly mimic the full metastatic cascade make research in this field challenging. In this Review, we discuss the most important mechanisms of immune system-metastasis interactions described to date, with the overall aim to underline their complexity and to highlight how they could be exploited therapeutically.

## Anti-tumor immunity

The cancer-immunity cycle, a complex sequence of interactions between multiple cell types, is required for the establishment of an efficient anti-tumor immune response (Chen and Mellman, 2013). To initiate this, cancer cells need to release tumor antigens, which are taken up by professional antigen-presenting cells, such as dendritic cells (DCs). When properly activated by local immuno-stimulatory signals, such as inflammatory cytokines or other factors released in the tumor microenvironment (TME), DCs migrate to tumor-draining lymph nodes and present tumor-derived antigens on MHC-I or MHC-II (see Glossary, Box 1) molecules to T cells. If T cells are, in turn, properly activated by DCs, they migrate to the tumor tissue and, upon recognition of their target antigen, induce cancer cell killing.

**Box 1. Glossary**

**γδ T cells:** ‘unconventional’ T cell subset that recognizes target antigens in a MHC-independent manner and expresses T cell receptors (TCRs) composed of γ and δ chains, as opposed to conventional helper and cytotoxic T cells, which express αβ TCRs.

**Anoikis:** programmed cell death induced by the loss of anchorage with the extracellular matrix.

**Colony-stimulating factor-1 (CSF-1):** also known as macrophage colony-stimulating factor-1, a cytokine that controls macrophage and monocyte lineage development, differentiation, survival and migration.

**Cyclo-oxigenases (COX):** enzymes that catalyze the synthesis of prostaglandins, which play crucial roles in the development of inflammation. COX1/2 enzymes are the target of nonsteroidal anti-inflammatory drugs (NSAIDs), such as aspirin.

**CD47:** transmembrane protein of the immunoglobulin family that is expressed on all the cells of the body. CD47 regulates various cellular functions, such as cell migration and cytokine production, but it is mainly known for its anti-phagocytic function and its function as a ‘don't eat me’ signal. Through the binding of SIRPα on myeloid cells, CD47 inhibits the phagocytosis of CD47-expressing cells. CD47 is frequently overexpressed by cancer cells of multiple tumor types.

**Diffuse-type giant cell tumor (Dt-GCT):** A rare proliferative disease affecting the joints that is characterized by CSF-1 overexpression. In the majority of Dt-GCT patients, CSF-1 overexpression is the result of a chromosomal translocation involving the gene encoding for CSF-1. This leads to massive recruitment of CSF-1R^+^ cells such as macrophages.

**Extracellular matrix (ECM):** an extracellular network of macromolecules including collagen, fibronectin and proteoglycans that regulates many aspects of cell behavior, such as cell-to-cell communication, cell adhesion, development and migration, both in healthy and in tumor tissue. The ECM is a highly dynamic structure that can be remodeled by proteases, such as MMPs and cathepsins. Tumors often overexpress ECM remodeling enzymes, and altered ECM dynamic contribute to cancer progression.

**Glucocorticoid-induced tumor necrosis factor receptor-related protein** **(GITR):** immune checkpoint molecule, expressed on effector and regulatory T cells, which plays a role in immunological self-tolerance.

**Granulocyte-colony stimulating factor (G-CSF):** a cytokine that regulates granulocyte development, differentiation, and recruitment.

**HLA-G:** a non-classical MHC-I molecule with multiple immunosuppressive functions, such as the ability to induce apoptosis and inhibit the cytotoxic activity of T and NK cells (Contini et al., 2003).

**Intercellular adhesion molecule 1 (ICAM1):** cell adhesion protein, expressed on endothelial and immune cells, that is involved in cell-cell interactions and leukocyte endothelial transmigration into tissues. ICAM1 binds to several integrins including CD11b.

**Interferon-γ (IFNγ):** a pro-inflammatory cytokine of the type II class of interferons with anti-viral and anti-tumor activities. It is produced mainly by NK and T cells.

**Interleukins (ILs):** cytokines or immunomodulatory mediators that can have pro- and anti-inflammatory functions.

**Major histocompatibility complex I and II (MHC-I and MHC-II):** a class of proteins that binds and presents peptides on the cell surface for recognition by T cells. MHC-I is expressed by all nucleated cells and presents peptides to CD8^+^ cytotoxic T cells. The downregulation of MHC-I expression by cancer cells is a frequent mechanism of tumor escape from T cell immune recognition. MHC-II is expressed by professional antigen-presenting cells, such as dendritic cells, macrophages and B cells, and presents peptides to CD4^+^ T cells. MHC molecules are fundamental for the development of adaptive immunity.

**Mast cells:** innate immune cells that store cytokines, chemokines, proteases and pro-angiogenic factors in large cytoplasmic granules. Mast cells are one of the main producers of histamine and play a crucial role in allergic responses, parasitic and bacterial infections and in cancer.

**Neutrophil extracellular trap (NET):** extracellular neutrophil-derived network of expelled DNA, fibers, histones and proteolytic enzymes. Release of NETs (NETosis) occurs in cases of pathogen infection, sterile inflammation and cancer.

**Pattern recognition receptor (PRR):** receptor that recognizes common pathogen-derived molecules and cell-derived danger signals called pathogen- and damage-associated molecular patterns, respectively. They play a role in activation of the innate immune system.

**Perforin:** glycoprotein produced mainly by cytotoxic T cells and NK cells. Upon release, perforin forms pores in the cell membrane of target cells and ultimately induces their lysis.

**Prostaglandin E2 (PGE2):** a bioactive lipid with a wide range of functions in inflammation and cancer. It is one of the downstream products of arachidonic acid metabolism by COX.

**Signal transducer and activator of transcription 3 (STAT3):** a transcriptional regulator expressed by many cell types including cancer and myeloid cells that promotes cell survival, proliferation and the secretion of pro-inflammatory factors. STAT3 activation in myeloid cells controls myeloid cell differentiation and recruitment as well as the production of immunosuppressive mediators, such as inducible nitric oxide synthase (iNOS), arginase 1 (Arg-1) and indoleamine-pyrrole 2,3-dioxygenase (IDO).

**Transforming growth factor β (TGFβ):** a cytokine that plays a dual role in cancer as it has both pro- and anti-inflammatory activities. TGFβ can function as a tumor growth suppressor, but it can also enhance tumor cell invasion and inhibit the function of immune cells. In cancer patients, TGFβ overproduction is frequently associated with metastasis and poor prognosis.

The spontaneous induction of effective anti-tumor immune responses that protect individuals from non-viral-induced tumors have been a matter of controversy (Dunn et al., 2002; Qin and Blankenstein, 2004). However, in the 1990s, several important pre-clinical and clinical observations supported the theory that the immune system controls some tumors. For instance, mice lacking T and B cells (Shankaran et al., 2001) or perforin-1 (Box 1) (Smyth et al., 1999; van den Broek et al., 1996) display a higher incidence and accelerated growth of immunogenic, chemically induced tumors. Moreover, the seminal work of Boon and colleagues, who identified tumor-specific antigens and T cells with specificity against these antigens in patients, proved that the adaptive immune system can detect cancer cells (Boon et al., 1994). The immune system's capacity to impair tumor growth is further supported by correlative studies showing a direct association between intratumoral infiltration of T cells and patient survival across different cancer types, including – but not limited to – ovarian, breast and colorectal cancer (Galon et al., 2006; Mahmoud et al., 2011; Zhang et al., 2003).

Clinical and experimental observations support the concept that the adaptive immune system may also protect against metastatic lesions. For instance, the depletion of CD8^+^ T cells in a spontaneous melanoma mouse model increased the formation of lung and reproductive tract metastases, demonstrating the anti-metastatic potential of T cells (Eyles et al., 2010; Lengagne et al., 2008). Besides T cells, natural killer (NK) and other immune cells may exert anti-metastatic functions (López-Soto et al., 2017). Indeed, pre-clinical studies report that defects in the NK cell compartment increase the risk of metastatic disease in mice (Smyth et al., 1998, 1999, 2000; Takeda et al., 2001). Moreover, an inverse correlation between the number of circulating or tumor-infiltrating NK cells and the presence of metastasis has been observed in patients with various solid tumors, such as colorectal and gastric cancer (Coca et al., 1997; Ishigami et al., 2000). Macrophages, neutrophils, eosinophils and mast cells can also mediate cancer cell killing directly through phagocytosis, production of reactive oxygen species, and secretion of cytokines, or indirectly by mediating the recruitment of T cells into the tumor through chemokine production (Benyon et al., 1991; Carretero et al., 2015; Cerwenka and Lanier, 2016; Chao et al., 2012; Katano and Torisu, 1982). However, as we discuss below, most studies on these myeloid immune cells point towards pro-metastatic functions.

In conclusion, the immune system can evoke an effective anti-tumor response (see poster, ‘Anti-tumor immunity’), but the sole presence of anti-tumor T cells or high NK cell numbers in cancer patients do not guarantee protective immunity, and the cancer-immunity cycle is frequently hampered in cancer patients. In the next sections, we discuss the mechanisms by which tumors escape from immune control.

## Tumor-promoting inflammation

One mechanism of immune evasion by tumors is the establishment of an immunosuppressive environment that inhibits the development or the efficacy of anti-tumor immune responses, both locally and systemically (see poster, ‘Immunosuppression’). Importantly, depending on the tumor type and stage, various immune cell populations contribute to the immunosuppressive environment. Immune cells exhibit considerable diversity and plasticity, and they respond to environmental signals by acquiring distinct functional phenotypes that can either inhibit or promote metastasis. Indeed, cancer cell-derived cytokines, such as transforming growth factor β (TGFβ) and interleukin (IL)-10 (Box 1), frequently skew the differentiation of tumor-infiltrating immune cells into a tumor-promoting phenotype (Flavell et al., 2010; Fridlender et al., 2009; Keirsse et al., 2017; Mantovani et al., 2002; Ondondo et al., 2013). These tumor-educated myeloid cells, particularly tumor-associated macrophages (TAMs) and neutrophils (TANs), can inhibit anti-tumor immune responses through the production of immunosuppressive cytokines, the expression of T cell co-inhibitory molecules, the consumption of amino acids that are crucial for the activity of effector T cells, and the production of reactive oxygen species (Fleming et al., 2018). Indeed, across almost all solid tumor types, a high neutrophil-to-lymphocyte ratio in the circulation is associated with poor survival (Templeton et al., 2014). Likewise, TAM abundance correlates with poor clinical outcome in several types of cancer (Campbell et al., 2011; Steidl et al., 2010).

The acquired phenotype of tumor-educated immune cells differs depending on cancer type and stage. For instance, neutrophils evolve from cytotoxic into tumor-promoting cells during tumor progression in mice bearing transplantable lung and mesothelial tumors (Mishalian et al., 2013). This observation is in line with clinical data showing that neutrophils isolated from surgically resected early-stage lung cancers were anti-tumorigenic and able to stimulate T cell proliferation and interferon-γ (IFNγ) (Box 1) release (Eruslanov et al., 2014). However, the bulk of experimental and clinical evidence supports the notion that neutrophils in advanced cancers sustain cancer progression and metastasis (Coffelt et al., 2016; Gentles et al., 2015; Templeton et al., 2014).

In addition to shaping immune cell functions, tumor-derived factors critically stimulate the recruitment and expansion of myeloid cells (see poster, ‘Recruitment of pro-tumorigenic immune cells’). Aberrant cytokine and chemokine production in the primary tumor drives the mobilization and recruitment of myeloid cells by manipulating their generation and release from the bone marrow (Casbon et al., 2015; Coffelt et al., 2015; Kitamura et al., 2015; Kowanetz et al., 2010). Hematopoietic stem and progenitor cells are frequently observed in the circulation of cancer patients compared with healthy controls, and these progenitors exhibit a myeloid bias toward granulocytic differentiation (Wu et al., 2014). This tumor-induced skewing of hematopoiesis and the systemic accumulation of myeloid cells has consequences for the immune composition of the TME and (pre-)metastatic organs. Indeed, TAMs are abundant in the TME of many cancer types (Gentles et al., 2015), and the circulation of cancer patients frequently contains expanded neutrophil populations (Templeton et al., 2014).

The aberrant cytokine production driving immune recruitment and polarization occurs by cancer cells directly, or through increased cytokine expression in the TME by, for instance, fibroblasts (Erez et al., 2010; Tauriello et al., 2018) and immune cells. Our group previously reported that TAMs in a spontaneous model of breast cancer produce IL-1β, which stimulates the expression of IL-17 (also known as IL-17A) from γδ T cells (Box 1). In turn, IL-17 induces granulocyte-colony stimulating factor (G-CSF, also known as CSF-3; Box 1) expression, which drives systemic neutrophil accumulation. These neutrophils acquire an immunosuppressive phenotype and promote metastasis by suppressing CD8^+^ T cells (Coffelt et al., 2015). Furthermore, in a spontaneous mouse model of mammary tumorigenesis, intratumoral CD4^+^ T cells produced cytokines, such as IL-4 and IL-13, which favor the activation and polarization of tumor-promoting immune cells, such as TAMs (Denardo et al., 2009).

Besides neutrophils and macrophages, other immune cells are implicated in tumor-promoting inflammation. DCs, which are required for the development of anti-tumor immune responses, can exert opposing functions and contribute to the generation of an immunosuppressive TME (Gabrilovich, 2004). The context in which DCs encounter antigens dictates their ability to trigger either immunity or tolerance (Keirsse et al., 2017). Tumor-derived factors interfere with the maturation of DCs, inducing a tolerogenic or immature phenotype characterized by low expression of co-stimulatory molecules and altered cytokine production both in patients and in mouse models (Almand et al., 2000; Dumitriu et al., 2009; Ghiringhelli et al., 2005; Labidi-Galy et al., 2011). Tolerogenic DCs inhibit anti-tumor T cell responses by impairing tumor antigen presentation, stimulating T cell exhaustion and inducing regulatory T cells (Tregs) (Dumitriu et al., 2009; Nakayamada et al., 2012). Depending on the cancer type, Tregs can make up a substantial proportion of tumor-infiltrating lymphocytes (Gentles et al., 2015). Although their immunosuppressive activity is crucial for preventing autoimmunity and excessive responses to pathogens, accumulation of Tregs and, in particular, a high ratio of Tregs versus effector T cells in tumor tissue, is associated with worse clinical outcome in the majority of solid tumors (Shang et al., 2015). Regulatory B cells (Bregs), a subpopulation of B cells with immunosuppressive functions, also contribute to tumor immune escape (Balkwill et al., 2013). A key function of Bregs is the conversion of CD4^+^ T cells into Tregs via IL-10 and TGFβ secretion (Olkhanud et al., 2011; Wang et al., 2015). Indeed, Breg deficiency reduced metastasis formation via loss of Treg cell conversion in transplantable breast cancer models (Olkhanud et al., 2011). Other pre-clinical studies have described Bregs as essential for myeloid cell polarization towards immunosuppressive phenotypes and subsequent metastasis formation (Bodogai et al., 2015). More recently, Breg expression of PD-L1 (also known as CD274) has been recognized as an important mediator of their immunosuppressive function, both in mouse models and invasive breast cancer patients (Guan et al., 2016; Zhao et al., 2018). Lastly, B cell-derived antibodies forming circulating immune complexes can promote cancer development by engaging activating Fcγ receptors on myeloid cells, which in turn regulate immune cell recruitment and function in the TME of human papilloma virus (HPV)-driven murine skin cancer lesions (Andreu et al., 2010). Whether immune complexes also play a role in metastasis is largely unknown.

In summary, the inhibition of the anti-tumor immune response through the orchestration of an immunosuppressive microenvironment is a crucial mechanism that contributes to cancer progression and metastasis. Importantly, tumor-educated immune cells can promote metastasis by additional means that go beyond the suppression of anti-tumor immunity. In the next sections, we address some of these mechanisms, and discuss how they may affect the metastatic cascade.

## Immune regulation of cancer cell invasion and intravasation

In order to metastasize, cancer cells need to egress from the primary site, invade the surrounding tissue and intravasate into blood or lymphatic vessels (see poster, panels 1 and 2, ‘Primary tumor growth’ and ‘Local invasion and intravasation’). Immune cells modulate these initial steps of the metastatic cascade by influencing extracellular matrix (ECM; Box 1) organization, vessel formation and permeability, and the motility of cancer cells. Different immune-mediated mechanisms are likely active or dominant in different tumor settings.

Immune cells are important regulators of the ECM, which affects many aspects of tumor biology (Lu et al., 2012). ECM remodeling can promote metastasis by different mechanisms: for instance, altered ECM dynamics favor tumor cell invasion by critically influencing the architecture of the surrounding tissue (Ghajar and Bissell, 2008), or by allowing the release and diffusion of growth factors and other pro-tumoral signaling molecules that are normally sequestered by the ECM (Cox and Erler, 2011). Various tumor-associated immune cells, but also fibroblasts and endothelial cells, influence the composition, organization and dynamics of the ECM by secreting ECM remodeling enzymes, such as matrix metalloproteinases (MMPs), cathepsins and other proteases. TAMs, TANs and mast cells (Box 1) can directly secrete ECM remodeling proteases (Gocheva et al., 2010; Nozawa et al., 2006; Ribatti et al., 2003), and Bregs can induce MMP expression in cancer cells (Ou et al., 2015). In mouse models of lung and skin cancer, immune cells are the main source of pro-tumorigenic and pro-metastatic MMPs (Acuff et al., 2006; Coussens et al., 2000). Noteworthy, the capacity of immune cells to remodel the ECM may also be beneficial for the development of an effective immune response: for instance, recent work in mice showed that the expression of specific ECM-remodeling enzymes in NK cells is crucial for their infiltration in lung metastatic lesions, and critically promoted immune control of the tumor (Putz et al., 2017).

Immune cells are also important regulators of angiogenesis and lymphangiogenesis (Alitalo et al., 2005; Ribatti and Crivellato, 2009). TAMs, mast cells and Tregs have been positively associated with blood vessel number or density in different types of tumors, including melanoma, pancreatic, breast and gastric cancer (Esposito et al., 2004; Leek et al., 1996; Yano et al., 1999; Zidlik et al., 2015). Blood and lymphatic vessels are essential for metastatic spread, because they represent the main routes for cancer cell migration to distant sites (Paduch, 2016). Additionally, blood vessels are a key supplier of nutrients and growth factors for the tumor. A dysfunctional vasculature, often the result of tumor-induced angiogenesis, is more permissive to tumor cell intravasation (Mazzone et al., 2009). It also affects the infiltration of immune cells in the TME, potentially preventing the action of the anti-tumor immune response (Hamzah et al., 2008). A major promoter of angiogenesis in the TME is hypoxia, caused by uncontrolled cell proliferation and concomitant lack of sufficient oxygen supply (Pugh and Ratcliffe, 2003). Hypoxia, in turn, influences the recruitment and function of tumor-infiltrating immune cells, promoting their pro-angiogenic functions (Kumar and Gabrilovich, 2014). For instance, TAMs can react to hypoxia by producing pro-angiogenic and lymphangiogenic factors, most importantly vascular endothelial growth factor A (VEGFA), which stimulates the proliferation and migration of endothelial cells (Ramanathan et al., 2003). Other tumor-infiltrating immune cells, such as Tregs and mast cells, can also produce VEGFA (Facciabene et al., 2011; Takanami et al., 2000). Moreover, CD4^+^ T cell-derived cytokines, such as IL-17, can promote the direct expression of VEGF in human cancer cells (Pan et al., 2015), and TAM and TAN-derived ECM remodeling enzymes, such as MMP9, can induce the release of pro-angiogenic factors that are trapped in the ECM (Lee et al., 2005). Interestingly, Tian and colleagues recently used transplantable mouse mammary tumor models and patient xenografts to show that intratumoral conventional CD4^+^ T cells contribute to vessel normalization, and that the vascular normalization gene expression signature correlated with T cell infiltration and activation in human breast and liver cancers (Tian et al., 2017). These results, which are in contrast to the pro-angiogenic role of CD4^+^ T cells, suggest that CD4^+^ T cells can potentially decrease tumor cell intravasation through a mechanism independent of the direct immune-mediated elimination of cancer cells.

Lastly, immune cells can directly promote tumor cell migration. For instance, work in a spontaneous mammary tumor model showed that IL-4/IL-13-activated TAMs, when exposed to tumor-derived colony-stimulating factor-1 (CSF-1; Box 1), produce epidermal growth factor (EGF), which in turn promotes motility and intravasation of EGF receptor (EGFR)-expressing tumor cells (Wyckoff et al., 2004). Noteworthy, intratumoral CD4^+^ T cells play a key role in this process, promoting the pro-metastatic TAM phenotype through IL-4 and IL-13 production (Denardo et al., 2009), which further highlights the dual role of CD4^+^ T cells in the early dissemination of cancer cells. Collectively, these data indicate how the intratumoral immune infiltrate strongly influences invasion and intravasation of cancer cells through a plethora of different mechanisms.

## Immune regulation of circulating tumor cell survival and extravasation

After intravasation, cancer cells need to survive in circulation, until they extravasate and colonize a distant site (see poster, panel 3, ‘Survival in circulation’). Circulating tumor cells (CTCs) endure a hostile environment, withstanding the shear stress of blood flow, death by anoikis (Box 1) and immune system attacks. A decreased cytotoxic functionality of NK cells correlates with increased numbers of CTCs in patients with metastatic breast, colorectal and prostate cancer (Green and Cruse, 2013; Santos et al., 2014), and liver-resident macrophages play a key role in trapping and eliminating CTCs (Denève, 2013), especially in the context of tumor-targeting antibody therapies (Gül, 2014). Furthermore, in patients with lung and breast cancer, the number of CTCs inversely correlates with the percentage of circulating T cells (Mego et al., 2016; Ye et al., 2017). However, the immune system can also promote CTC survival. For instance, CTCs directly interact and associate with platelets, which promotes their activation (Camerer et al., 2004). Consequently, activated platelets form a fibrin clot around CTCs, which promotes CTC survival by shielding them from NK cell attack (Palumbo et al., 2005). Moreover, Tregs can directly provide survival signals to CTCs through the production of receptor activator of nuclear factor-B ligand (RANKL) (Tan et al., 2011). CTCs can employ additional strategies to escape immune control. For instance, CTCs in colorectal cancer patients were shown to upregulate the anti-phagocytic molecule CD47 (Box 1), compared with matched primary tumors (Steinert et al., 2014). Moreover, researchers observed increased levels of secreted HLA-G (Box 1) in the blood of breast cancer patients compared with healthy controls, and that the number of CTCs in patients directly correlates with the level of secreted HLA-G (König et al., 2016). This suggests that HLA-G-induced immunosuppression mechanisms are important for the survival of CTCs. Finally, the presence of CTCs in breast cancer patients associates with an increased percentage of FAS-expressing T cells. Upon triggering by its ligand, FASL, FAS induces T cell apoptosis (Gruber et al., 2013). Noteworthy, the aforementioned immune escape strategies used by CTCs provide circumstantial evidence for the role of NK and T cells in eliminating CTCs; further work is needed to provide insights on the mechanisms used by these immune cells to eliminate CTCs from the flowing blood.

Although CTCs employ several strategies to survive the hostile environment of the circulation, their metastatic potential eventually depends on their ability to extravasate and grow out in distant organs (see poster, panel 5, ‘Extravasation’). For extravasation, cancer cells need to adhere to the endothelium and modulate it so they can migrate through and invade into the surrounding tissue at a distant site. Tumor cells can be physically restrained in small capillaries, but extravasation from larger vessels requires active adhesion processes that immune cells can contribute to. For instance, neutrophils release neutrophil extracellular traps (NETs; Box 1) that trap CTCs in distant organs and stimulate their invasion and expansion (Cools-Lartigue et al., 2013; Park et al., 2016). The interaction of neutrophil integrin CD11b (also known as ITGAM) with intercellular adhesion molecule 1 (ICAM-1; Box 1) on cancer cells (Spicer et al., 2012) and platelet-induced clot formation (Stegner et al., 2014) further promote tumor cell adhesion. Platelets also contribute to extravasation by promoting an invasive mesenchymal-like phenotype in cancer cells through direct platelet-cancer cell interactions and through the release of TGFβ (Labelle et al., 2011), or by releasing ATP-containing granules that modulate the endothelial lining and cause vascular leakiness (Schumacher et al., 2013).

In addition to neutrophils and platelets, recruitment of monocytes to extravasation sites enhances cancer cell survival and extravasation. *Ex vivo* imaging studies in experimental lung metastasis models have demonstrated that inflammatory monocytes aid extravasation by physically associating with cancer cells as they migrate through the endothelial lining (Qian et al., 2009). Additionally, inflammatory monocytes promote vascular permeability and subsequent metastatic seeding through VEGF production in an orthotopic breast cancer model (Qian et al., 2011). These inflammatory monocytes differentiate into immunosuppressive precursors of metastasis-associated macrophages that suppress CD8^+^ T cells upon recruitment into metastatic sites in mouse models of breast cancer (Kitamura et al., 2018).

Although the studies described above were conducted in different models of metastasis and identify distinct mechanisms, it is likely that various cells work together to promote extravasation and survival. Indeed, some studies have reported crosstalk between platelets, endothelial cells and macrophages, as well as neutrophils (Chen et al., 2011). Thus, although the immune system contributes to the clearing of CTCs, it can also promote their survival and mediate their extravasation. Once more, the balance between pro-tumoral inflammation and anti-tumor immunity crucially determines the outcome of these steps of the metastatic cascade.

## The (pre-)metastatic niche: immune cells as key coordinators of ‘fertile soil’

The processes that regulate and determine the sites where metastases will form have been debated for decades. In 1889, Stephen Paget proposed the ‘seed and soil’ hypothesis, which postulates that the distribution of metastases is not random; instead, cancer cells (the ‘seeds’) colonize preferentially those organs in which the environment is favorable (the ‘congenial soil’) (Paget, 1889). Paget's view was challenged in the late 1920s by James Ewing, who argued that organotropism could be explained solely by the design of the circulatory system (Ewing, 1928). This remained the dominant view until the 1970s, when Isaiah Fidler provided the first experimental evidence to support Paget's hypothesis by demonstrating that, although the design of the circulatory system is important, certain cancer types only colonize particular organs (Fidler and Nicolson, 1976). In the following years, studies addressing the determinants of organotropism focused mostly on tumor-intrinsic properties. For instance, human breast cancer cells frequently express the chemokine receptors CXCR4 and CCR7, which have been reported to guide their spread to the bone, lung and regional lymph nodes that express high levels of the ligands CXCL12 and CCL21, respectively (Müller et al., 2001). It became apparent in recent years that factors extrinsic to the tumor, in particular stromal and immune cell populations, are equally important determinants for metastatic spread. Tumors actually prepare distant organs for the arrival of disseminated cancer cells by inducing a number of systemic molecular and cellular changes that create a supportive and receptive microenvironment for colonization, referred to as the pre-metastatic niche (see poster, panel 4, ‘The (pre)metastatic niche’).

In a pioneering paper that emphasized the significance of immune cells in the formation of the (pre-)metastatic niche, Kaplan and colleagues demonstrated that VEGF receptor 1-expressing (VEGFR1^+^, also known as FLT1) bone marrow-derived cells (BMDCs) are recruited to the lung in response to tumor-derived VEGFA and placental growth factor before the arrival of transplanted tumor cells (Kaplan et al., 2005). The BMDCs established a permissive niche for incoming tumor cells through expression of chemoattractants. This was the first study to demonstrate how immune cells orchestrate the site of future metastases. Later studies shed more light on how an intimate crosstalk between primary tumor-derived factors, the local stromal microenvironment and BMDCs regulates the formation of the (pre-)metastatic niche (Liu and Cao, 2016). As the primary tumor grows and becomes more hypoxic and inflammatory, increased secretion of tumor-derived factors (Liu and Cao, 2016; Peinado et al., 2017) and extracellular vesicles (Hoshino et al., 2015; Peinado et al., 2012) stimulates the mobilization and recruitment of (immature) myeloid cells directly from the bone marrow, thereby initiating the (pre-)metastatic niche. These factors also induce changes in the stromal compartment of the distant organ that support the influx of BMDCs and CTCs (Erler et al., 2009; Hiratsuka et al., 2008). Continuous influx of BMDCs further remodels the local environment into a tumor-promoting (pre-) metastatic niche characterized by increased angiogenesis and vascular permeability, ECM remodeling, chronic inflammation and immunosuppression (Liu and Cao, 2016).

Neutrophils, macrophages and fibroblasts have been identified as the main sources of proangiogenic molecules like Bv8 (also known as PROK2), as well as of ECM remodeling factors, such as fibronectin and lysyl oxidase (LOX). These facilitate tumor cell recruitment and extravasation in the (pre-)metastatic niche (Erler et al., 2009; Hiratsuka et al., 2002; Kowanetz et al., 2010; Qian et al., 2011; Yamamura et al., 2015). Chronic inflammation in the (pre-)metastatic niche is an important driver of metastasis by promoting the recruitment of both BMDCs and tumor cells to distant organs (Wang et al., 2017; Wculek and Malanchi, 2015). For instance, blocking pro-inflammatory molecules or their receptors suppressed the recruitment of myeloid cells to the pre-metastatic niche and, in turn, hampered metastasis formation in Lewis lung carcinoma and B16 melanoma models (Hiratsuka et al., 2006, 2008). Lastly, the establishment of an immunosuppressive microenvironment is an essential characteristic of the (pre-) metastatic niche. It allows cancer cells to escape immune recognition and progress to form macro-metastases (Clever et al., 2016; Sceneay et al., 2013). Our group and others have shown that tumors induce systemic accumulation of immunosuppressive neutrophils that promote metastasis by suppressing CD8^+^ T cell responses (Coffelt et al., 2015; Giles et al., 2016), highlighting immunosuppression as an essential feature of metastasis formation.

Although it is clear that establishment of a supportive immunosuppressive environment greatly favors metastatic seeding (see poster, panel 6, ‘Colonization and persistent growth’), whether we can truly distinguish the pre-metastatic niche from the metastatic niche is debatable, because providing evidence of the absence of cancer cells in the (pre-)metastatic niche is challenging. Moreover, many of the changes induced by growing tumors are systemic in nature, not limited to organs in which metastases will develop. Some elements of the (pre-)metastatic niche that have been described in mice can be found in clinical samples. These include neutrophil accumulation in the blood (Templeton et al., 2014), clusters of VEGFR1^+^ cells detected in common sites of metastasis before tumor spread (Kaplan et al., 2005), elevated MMP9 levels in the lungs of patients with distant tumors (Hiratsuka et al., 2002), and LOX and CD11b^+^ cells in metastatic tissues (Erler et al., 2009). Although this evidence is indirect, from a clinical perspective, it would be very interesting to identify biomarkers that could identify cancer patients with signs of pre-metastatic niche formation, and to treat these patients with pre-metastatic niche-disrupting agents.

## Inhibiting tumor-promoting inflammation to fight metastatic disease

Inhibiting tumor-promoting inflammation is an appealing strategy to fight metastasis. Given the clear immunosuppressive and pro-metastatic roles of tumor-infiltrating macrophages, neutrophils, and Tregs, researchers are extensively exploring strategies targeting their recruitment, polarization, and effector molecules (see poster, ‘Recruitment of pro-tumorigenic immune cells’). Noteworthy, the effects of targeting pro-tumor immune cells can go beyond the activation of anti-tumor immune responses. For instance, depletion/repolarization of TAMs might affect their pro-angiogenic and ECM remodeling functions as well as their immunosuppressive functions (Gazzaniga et al., 2007). Although this line of research is relatively new, the oncology field can adopt the cytokine and receptor inhibitors that are FDA approved for the treatment of chronic inflammatory and autoimmune diseases.

Receptors that mediate the recruitment of pro-metastatic immune cells and their respective ligands are potentially important targets for the treatment of metastasis. Among these, perhaps the most explored approach to date is targeting the CSF-1/CSF-1R pathway that mediates macrophage infiltration (Ries et al., 2014). In a mouse model of spontaneous breast cancer, researchers showed that genetic ablation of CSF-1 does not affect primary tumor incidence or growth, but strongly inhibits the development of metastases through a reduction in TAMs (Lin et al., 2001). Therapeutic targeting of this axis inhibited metastasis formation in mouse models of breast and pancreatic cancer (Mitchem et al., 2013; Ruffell et al., 2014). Preliminary results of a clinical trial with a monoclonal antibody against CSF-1R showed a reduction in TAMs and an increase in CD8^+^/CD4^+^ T cell ratio in patients with various solid tumors, and led to good objective responses in diffuse-type giant cell tumor patients (Dt-GCT; Box 1) (Ries et al., 2014). This provided the first evidence for the clinical benefit of CSF-1/CSF-1R pathway targeting for the treatment of cancer.

Similarly, disruption of neutrophil recruitment through CXCR2 blockade prevented metastasis formation in cell line, xenograft and spontaneous models of rhabdomyosarcoma, colon and pancreatic cancer (Highfill et al., 2014; Steele et al., 2016; Wang et al., 2017), and several CXCR2 inhibitors are currently under clinical investigation. The dominant pathway for Treg recruitment is through tumor- or TAM-derived CCL22 that binds CCR4 on Tregs, providing rationale for CCR4 antagonists (Curiel et al., 2004; Kurose et al., 2015; Sun et al., 2017).

The upstream soluble mediators that drive pro-metastatic immune cell expansion and accumulation could also be potential targets. A candidate signaling pathway to prevent neutrophil accumulation is the IL-1β–IL-17–G-CSF axis (Coffelt et al., 2015). Tregs expand in response to TGFβ or elevated levels of prostaglandin E2 (PGE2; Box 1) (Olkhanud et al., 2011; Yang et al., 2012), arguing for TGFβ neutralization or inhibitors of cyclo-oxigenases (COX; Box 1) as potential targets (Karavitis et al., 2012). Moreover, stromal fibroblast-derived TGFβ is an important driver of immune evasion and metastasis and conveys resistance to PD-L1 blockade in a mouse model of spontaneous colon cancer metastasis and in metastatic urothelial cancer patients (Mariathasan et al., 2018; Tauriello et al., 2018).

The CCL2/CCR2 interaction could be an attractive target to prevent monocyte recruitment, but clinical trial results have been inconsistent (Kitamura and Pollard, 2015; Pienta et al., 2013). CCL2 neutralization inhibited metastasis formation by retaining monocytes in the bone marrow in spontaneous breast and pancreatic cancer models of metastasis (Mitchem et al., 2013; Qian et al., 2011), but cessation of treatment caused a compensatory influx of monocytes into metastatic sites and resulted in increased mortality (Bonapace et al., 2014). Relatedly, depending on the tumor characteristics, treatment with single chemokine/receptor antagonists might not be sufficient to suppress metastasis formation, as ligand and receptor redundancy allows recruitment of specific cell types (Coffelt et al., 2016; Kitamura and Pollard, 2015). Moreover, several immune cell types may support metastasis formation – targeting one could lead to a compensatory increase and dependency on the other. Indeed, inhibition of TAMs through CSF-1R targeting led to neutrophil accumulation and enhanced metastasis formation in transplantable mouse models of melanoma, lung, colon and breast cancer (Kumar et al., 2017; Swierczak et al., 2014). In the Lewis lung carcinoma model of spontaneous metastasis, CSF-1R inhibition unexpectedly promoted metastasis formation by indirectly diminishing NK cell numbers through removal of the TAM-derived NK cell survival signal IL-15 (Beffinger et al., 2018). These studies emphasize the complexity of targeting the dynamic cancer-immune cell interactions, especially with the myeloid compartment.

Instead of inhibiting the recruitment of specific immune cells, repolarizing immune cells from a pro-tumorigenic to anti-tumorigenic phenotype might be a preferred strategy. For instance, TAMs could be re-educated into an anti-tumor phenotype by CSF-1R inhibition or by pattern recognition receptor (PRR; Box 1) ligands, which inhibited tumor growth in a mouse model of glioblastoma multiforme (Pyonteck et al., 2013) and lung cancer (Shime et al., 2012), respectively. Similarly, TGFβ blockade reverted immunosuppressive neutrophils into a cytotoxic phenotype (Fridlender et al., 2009). Re-programming of the immunosuppressive microenvironment might also be achieved through signal transducer and activator of transcription 3 (STAT3; Box 1) inhibition (de Haas et al., 2016; Kroemer et al., 2016). Tregs might be targeted by glucocorticoid-induced tumor necrosis factor receptor-related protein (GITR; Box 1) agonists, which were shown to convert Tregs into anti-tumor effector T cells that have lost immunosuppressive functions in a melanoma model (Schaer et al., 2013). Whether these changes in immune cell phenotype affect metastasis formation requires further investigation.

Finally, targeting the immunosuppressive mediators and cytokines in the TME has shown promise as well. Neutralization of TAM-derived IL-10 enhanced T cell responses to a similar extent as TAM depletion using anti-CSF-1 antibodies in a spontaneous breast cancer mouse model (Ruffell et al., 2014).

Because the availability of drugs that specifically target pro-tumor immune cells is still limited, an alternative approach is to use conventional anti-cancer therapies with immunomodulatory properties, such as chemotherapy, radiotherapy and oncogene-targeted therapies (Kersten et al., 2015; Wargo et al., 2015). These approaches can directly deplete certain pro-tumor immune populations (Han et al., 2012; Lutsiak et al., 2005), or indirectly activate anti-tumor immune response through increased antigen availability and induction of immunogenic cell death (Galluzzi et al., 2015).

Although the different strategies discussed in this section might be effective in alleviating pro-tumor inflammation, it is likely necessary to combine strategies targeting the immunosuppressive TME with therapies aimed at boosting the anti-tumor immune response (Box 2), in order to maximize therapeutic benefit. A growing body of pre-clinical evidence supports this approach. For instance, CXCR2 blockade improved the response to checkpoint blockade in transplantable rhabdomyosarcoma and spontaneous pancreatic cancer models (Highfill et al., 2014; Steele et al., 2016). Furthermore, CSF-1R inhibition enhanced the efficacy of adoptive T cell transfer in transplantable melanoma models (Mok et al., 2014). Although some of the aforementioned strategies still await verification in the clinical setting, several approaches are currently in early clinical trials evaluating their safety and tolerability, and measurements of secondary outcomes including tumor-infiltrating immune cells or cytokine and chemokine serum levels will provide the first indication of the therapeutic value of these approaches.

**Box 2. Boosting anti-tumor immunity to fight metastatic disease**

The immune system is tightly intertwined with the metastatic cascade, and the balance between pro-tumor inflammation and anti-tumor immunity is a key factor in metastasis. Shifting the balance towards anti-tumor immunity therefore represents an attractive therapeutic approach to prevent or cure metastasis (see poster, ‘Anti-tumor immunity’).

A promising strategy to boost anti-tumor immune responses is the use of immune checkpoint inhibitors, which target T cell-inhibitory molecules, such as PD-1 and CTLA-4. Although the exact mechanism is still debated, blocking PD-1 (or its ligand PD-L1) and CTLA-4 improved the survival of patients with advanced metastatic melanoma and with other solid tumors to an extent that no other therapeutic strategy, including non-immune based approaches, ever reached (Hodi et al., 2010; Overman et al., 2017; Topalian et al., 2012). The striking clinical efficacy of immune checkpoint inhibitors for advanced metastatic cancers represented a crucial breakthrough and fueled the acknowledgment of immunotherapy as one of the pillars of cancer therapy. In addition to metastatic melanoma, antibodies targeting inhibitory or immune checkpoint molecules are now FDA approved for the treatment of metastatic non-small cell lung cancer, renal cell carcinoma, and head and neck squamous cell carcinoma, and for all metastatic solid tumor types with high microsatellite instability or DNA mismatch repair deficiency (Alexander, 2016). That said, only a proportion of patients respond to immune checkpoint inhibitors (Larkin et al., 2015), and the efficacy of this approach against other – less immunogenic – metastatic cancers is expected to be less spectacular and might require combination strategies.

Another approach to exploit the immune system to fight metastatic disease is adoptive cell transfer (ACT) of specific anti-tumor T lymphocytes into patients, after *ex vivo* expansion, activation and, sometimes, genetic modification (Humphries, 2013; June et al., 2018). The use of autologous T cells that recognize antigens derived from somatic mutations in cancer cells proved effective for the treatment of patients affected by metastatic melanoma, breast, colorectal and bile duct cancers (Tran et al., 2014, 2016; Yang and Rosenberg, 2016; Zacharakis et al., 2018). This technology thus represents a promising therapeutic approach for metastatic solid tumors.

An alternative to ACT, which overcomes the hurdle of identifying and isolating tumor-specific T cells for each patient, is to activate endogenous anti-tumor T cells directly *in vivo.* This can be achieved by, for instance, exploiting the central role for DCs in kick-starting anti-tumor immunity. PRR (Box 1) ligands can promote the activation and maturation of intratumoral DCs (Goutagny and Fitzgerald, 2006; Goutagny et al., 2012), but their anti-metastatic effect has so far been reported only for skin lesions (Dummer et al., 2008). Alternatively, antibodies that target immuno-stimulatory molecules, such as CD40 (Byrne and Vonderheide, 2016; Vonderheide and Glennie, 2013), can also promote the activation of DCs. However, the benefit of CD40 agonists as monotherapy for the treatment-advanced solid tumors was modest in early-phase clinical trials (Johnson et al., 2015; Rüter et al., 2010; Vonderheide et al., 2007).

A different and tumor-specific DC-based immunotherapy involves the isolation of patient- or donor-derived DCs, or DC precursors, and their maturation and activation *ex vivo*. DCs are then exposed to tumor-derived antigens, such as bulk cancer cell lysate, tumor antigen-derived peptides or mRNAs coding for tumor antigens, and re-infused in patients. An example of this approach is Sipuleucel-T, a vaccine formulated to include patient-derived antigen-presenting cells that are activated and exposed to tumor antigens *ex vivo*. Sipuleucel-T was FDA approved in 2010 for the treatment of metastatic castration-resistant prostate cancer (Kantoff et al., 2010). In addition, DCs can be targeted *in vivo* through vaccination, administrating tumor-derived peptides (van Poelgeest et al., 2013), or DNA- or mRNA-encoding tumor-derived antigens. Recent work showed that vaccination with RNA coding for neo-antigens of patients with advanced melanoma induced a specific anti-tumor T cell response, promoted the infiltration of neo-antigen-specific T cells in the metastatic lesions and led to objective responses in a significant proportion of the patients (Sahin et al., 2017), illustrating the potential of this approach.

Stimulatory cytokines can also promote an anti-tumor T cell response: IL-2, for example, promotes the expansion of T cells, and its administration to metastatic melanoma and renal carcinoma patients produced remarkable clinical benefit (Rosenberg et al., 1994). However, its use is limited by severe immune-related side effects (Ahmadzadeh and Rosenberg, 2005). Currently, researchers are exploring different strategies to reduce IL-2 toxicity – for example, by decreasing its receptor-binding affinity or increasing its half-life, allowing in turn the use of lower doses (Choudhry et al., 2018) – but their efficacy and safety in clinical settings remains to be evaluated.

Besides T cells, targeting NK cells represents a promising approach for treatment of metastatic patients. NK cells can be pharmacologically targeted using antibodies that block NK cell inhibitory receptors or trigger activating receptors (Guillerey et al., 2015). Moreover, stimulating cytokines such as IL-2 and IL-15 can promote NK cell functionality (Romee et al., 2014). Finally, NK cells can be adoptively transferred to patients, but so far this has had only limited therapeutic efficacy, despite promising pre-clinical results (Parkhurst et al., 2011).

Compared with T cells, NK cells and DCs, there have been fewer attempts to directly enhance the anti-tumor activity of myeloid cells such as macrophages and neutrophils, mainly because the majority of myeloid cells have immunosuppressive or pro-tumor functions in the TME. One exception is the use of antibodies targeting CD47, a ‘don't eat me’ signal often overexpressed on malignant cells (Matlung et al., 2017). The antibody binding of CD47 promotes tumor cell phagocytosis by macrophages, neutrophils and DCs (Willingham et al., 2012). The efficacy of this approach is currently under evaluation in clinical trials (Weiskopf, 2017).

In conclusion, the recent and impressive clinical responses of cancer patients treated with immune checkpoint inhibitors highlight the potential of cancer immunotherapy for the treatment of advanced metastatic disease. However, boosting the immune system might not be enough: considering all immune-boosting strategies described here, their anti-metastatic effect, when applied as monotherapy, is often limited to a proportion of the patients, and to few tumor types. Inhibiting pro-tumoral inflammation represents a promising and complementary strategy to improve the efficacy of immunotherapy for metastatic disease.

## Conclusion and perspective

The recent achievements of immunotherapy for the treatment of advanced metastatic cancers (Box 2) have encouraged many clinical trials assessing the efficacy of these approaches in different tumor types, alone and in combination with conventional therapies. Notwithstanding the clinical successes of cancer immunotherapy, clinical trials are currently outpacing our scientific understanding of the immune-related mechanisms that influence metastasis formation and response to therapy. Several challenges remain. Tumors of different types, and even individual tumors of the same type, vary greatly in their immune landscape, both locally and systemically (Gentles et al., 2015), and the reasons behind this heterogeneity are still largely unexplained. Moreover, the different factors that regulate the sensitivity of organ-specific metastases versus primary tumors to immunomodulation are unknown. For instance, differences in metabolism, genetic makeup or epigenetics between cancer cells in the primary tumors versus metastases are likely to contribute to their differential sensitivity to immunotherapy. A fundamental understanding of these unresolved issues is essential to make rational decisions about which patients to select, to tackle therapy resistance and design the most optimal therapy combinations, and thus maximize the therapeutic benefit of immunomodulatory strategies. The use of spontaneous mouse tumor models that closely recapitulate the different metastatic steps, and in-depth characterization of the immune landscape in those models, in combination with comprehensive immune monitoring in cancer patients will be critical to rationally design the most efficacious immunotherapy strategies to tackle metastases in individual patients, striving towards personalized immune interventions.

## Supplementary Material

Poster
