# Supplementary figures and images for: Immune regulation of metastasis: mechanistic insights and therapeutic opportunities
Source: Dis Model Mech. 2018 Oct 24;11(10):dmm036236. doi: 10.1242/dmm.036236 (PMC6215427; doi:10.1242/dmm.036236)

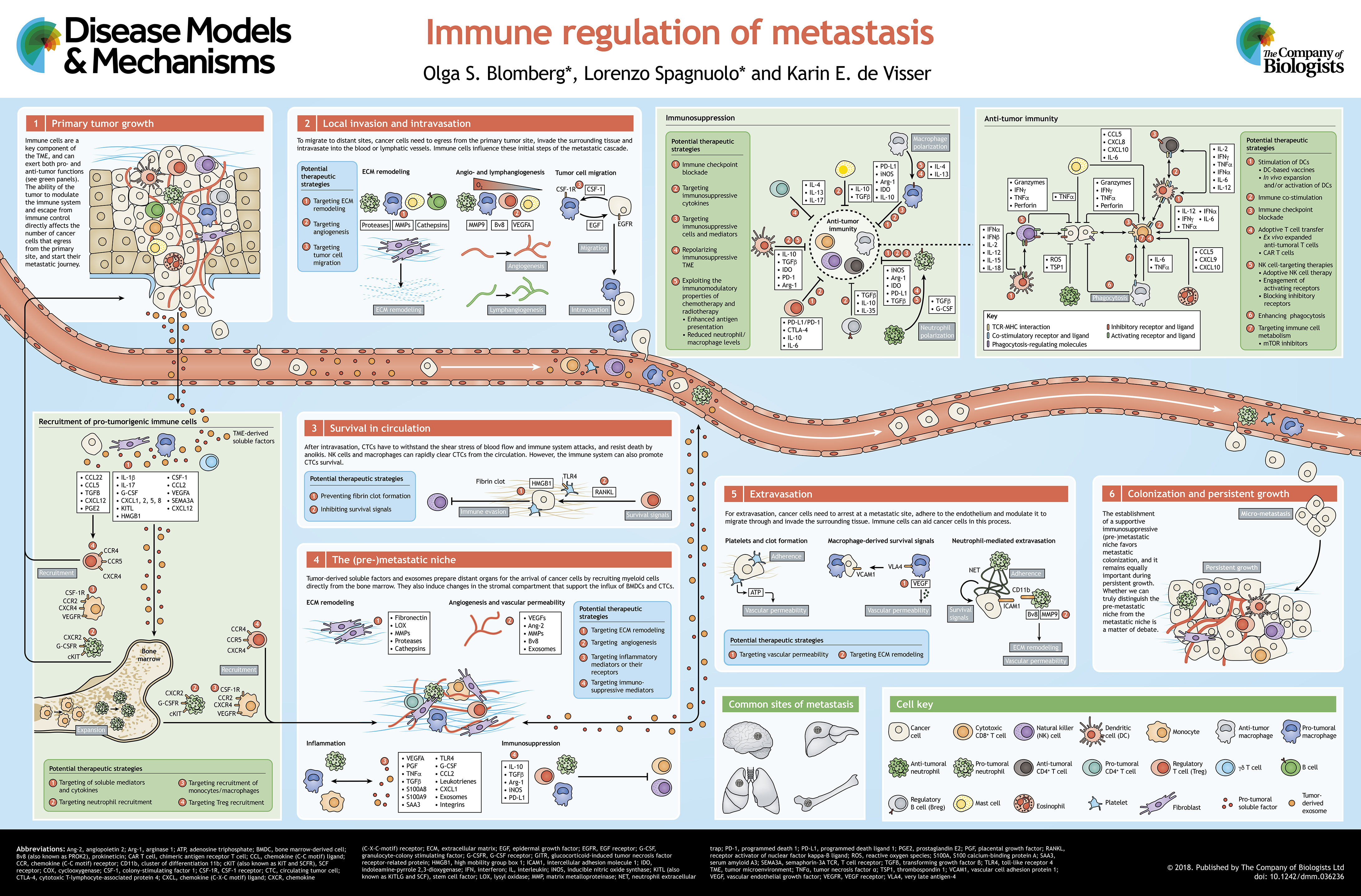

Supplement: Poster [file dmm-11-036236-s1.jpg]
